# Oral food challenge outcomes in a pediatric tertiary care center

**DOI:** 10.1186/s13223-017-0215-8

**Published:** 2017-09-22

**Authors:** Elissa M. Abrams, Allan B. Becker

**Affiliations:** Department of Pediatrics, Section of Allergy and Clinical Immunology, University of Manitoba, FE125-685 William Avenue, Winnipeg, MB R3E 0Z2 Canada

**Keywords:** Food allergy, Oral food challenge, Anaphylaxis

## Abstract

**Background:**

Oral food challenges are the clinical standard for diagnosis of food allergy. Little data exist on predictors of oral challenge failure and reaction severity.

**Methods:**

A retrospective chart review was done on all pediatric patients who had oral food challenges in a tertiary care pediatric allergy clinic from 2008 to 2010.

**Results:**

313 oral challenges were performed, of which the majority were to peanut (105), egg (71), milk (41) and tree nuts (29). There were 104 (33%) oral challenge failures. Children were more likely to fail an oral challenge if they were older (P = .04), had asthma (P = .001) or had atopic dermatitis (P = .03). Risk of challenge failure was significantly different between food allergens, with more failures noted for peanut than for tree nuts, milk or egg (P = .001). Among challenge failures, 19% met criteria for anaphylaxis. Significantly more tree nut and peanut challenges met criteria for anaphylaxis than milk or egg (P < .001). Skin test size and specific IgE level were significantly higher in those who failed oral challenges (P < .001). The highest rate of challenge failure and severity of failure was to cashew, with 63% of cashew challenges reacting, of which 80% met clinical criteria for anaphylaxis.

**Conclusion:**

The risk of challenge failure differed with type of food studied, with peanut and tree nut having a higher risk of challenge failure and anaphylaxis. Cashew in particular carried a high risk and caution must be exercised when performing these types of oral challenges in children.

## Background

Food allergy affects 2–10% of the population, and is more common in children than adults [1]. The diagnosis of food allergy is often based on results of a careful history, skin prick testing (SPT) and serum food-specific IgE [2]. Oral food challenges (OFCs) assist in the diagnosis of food allergy, and are essential to determine whether an allergy has been outgrown [3]. However, OFCs do carry the risk of a systemic allergic reaction [3]. While the double blind placebo controlled food challenge is the most accurate and a true ‘gold standard’ for diagnosis of food allergy, it is time consuming and costly [3]. The open oral food challenge is often used instead, although it is subject to patient bias [3].

Previous studies have examined the feasibility and safety of oral food challenges, as well as diagnostic levels at which to consider food challenges based on results of serum food-specific IgE and/or epicutaneous testing [4–10]. However, there is a paucity of literature examining other predictors of food challenge outcomes.

We performed a retrospective chart review to examine whether oral food challenge outcomes varied by characteristics such as food being challenged, patient characteristics (age, atopy), and results of skin prick testing or serum food-specific IgE.

## Methods

A retrospective chart review was performed on all open oral food challenges between January 1, 2008 and December 31, 2010 at the University of Manitoba pediatric allergy clinic. Oral food challenges were performed based on the clinical decision of the attending physician, with consideration of clinical history, results of epicutaneous testing, and/or results of serum food-specific IgE. Challenges were performed to confirm food allergy, or when there was a suspicion of oral tolerance after a period of avoidance in a food allergic child.

This study met the criteria for a waiver of informed consent by the research ethics board at the University of Manitoba as it was an internal quality improvement study.

The oral challenge was administered as half-log (base 10) incremental doses (starting at .1 mg for solids and .1 mL for liquids) every 15–20 min until a final dose of 10 g (30–100 mL for cows’ milk) was tolerated. In children with asthma, oral challenges were only performed if asthma was well controlled. Challenges were terminated and considered positive if there were objective symptoms noted by the attending physician, or, on occasion, when only worrisome subjective symptoms (subjective oropharyngeal symptoms) were reported by the patient. Patients were observed for an hour after the final dose. If there was a reaction, patients were observed for a minimum of an hour, until objective signs of the reaction had resolved. Patients were asked to notify the attending physician should there be any delayed reaction after discharge.

Treatment of challenge failures was at the discretion of the attending physician, and based on reaction severity. If the patient met the criteria for anaphylaxis, .01 mg/kg of intramuscular epinephrine (1:1000) was administered. A repeat dose was given in 10–15 min if there was no symptom resolution. Other treatment of positive challenges was at the discretion of the attending physician and included an age-appropriate dose of antihistamine for cutaneous symptoms, 2.5–5 mg inhaled albuterol for respiratory symptoms refractory to epinephrine, and an age appropriate dose of prednisone (.1 mg/kg).

### Statistical methods

Statistical analyses were performed by using SAS version 9.3 (SAS Institute, Cary NC). Pearson’s Chi square test was used for categorical variables, Kruskal–Wallis test was used for comparing continuous distributions between groups, and relative risk was used as a measure of association. P < .05 was considered to be statistically significant.

## Results

There were 313 oral food challenges performed between January 1, 2008 and December 31, 2010 at the University of Manitoba Pediatric Allergy Clinic. There were 105 peanut, 71 egg, 41 milk, 29 tree nut (6 almond, 1 brazil nut, 8 cashew, 6 hazelnut, 1 macadamia nut, 2 pecan, 5 walnut), 10 finned fish, 14 shellfish, 9 soy, and 34 other challenges performed. Seventeen patients underwent oral challenges to more than one food during this time (although never more than one food each day), and eleven patients had more than one oral challenge to the same food. Some peanut and tree nut challenges were masked (often in pudding).

Table 1 shows the characteristics of the study population. There were 104 oral food challenge failures (33% of food challenges), of which 82 were objective and 22 were subjective failures (predominantly subjective oropharyngeal symptoms).

**Table 1:** Patient demographics of failed versus passed oral challenges

	Passed	Failed	Total	P value
Median age (months)	58	73	66	.04
Female (%)	43	36	40	–
Overall atopy (%)	72	79	74	.16
Atopic dermatitis (%)	60	74	65	.03
Asthma (%)	47	72	55	.001
Multiple food allergy (%)	49	46	48	.62
Aeroallergen sensitization (%)	81	81	81	.96

Median patient age was 5.5 years (range 8 months–18 years). Older children were significantly more likely to fail an oral challenge than younger children (median age 73 months vs 58 months; P = .04). There was no difference in overall rate of atopy (defined as atopic dermatitis, other food allergy, asthma, or aeroallergen sensitization) between those who failed and those who passed oral challenges. Overall rate of other atopic disease was high at 74%. Rate of physician diagnosed atopic dermatitis was significantly higher among those who failed oral challenges (60% vs 74%; P = .03). Rate of asthma was also significantly higher among those who failed oral challenges (47% vs 72%; P = .001). Rate of multiple food allergy and aeroallergen sensitization were not significantly different among those who failed oral challenges.

Clinical characteristics of challenge failures are noted in Table 2. Risk of challenge failure was significantly different between food allergens (P = .001), with more failures noted for peanut than for tree nut, milk or egg (P = .001). Among challenge failures, 20/104 (19%) met the criteria for anaphylaxis (epinephrine administration or multi-organ involvement). Significantly more tree nut and peanut challenges met the criteria for anaphylaxis than milk or egg (P < .001). There were no documented incidences of biphasic reactions and no reactions that included hypotension or required hospital admission.

**Table 2:** Clinical characteristics of challenge failures

	Milk	Egg	Peanut	Tree nut	Total	P value
Challenge failures (% per food)	34	28	47	34	33	.001
Anaphylaxis (%)	7	5	20	70	19	<.001
Urticaria (%)	29	55	55	70	52	.31
Angioedema (%)	0	0	12	30	11	.06
Gastrointestinal symptoms (%)	29	5	14	30	14	.11
Respiratory symptoms (%)	0	0	0	40	4	<.001
Subjective symptoms (%)	36	40	8	0	21	.001

The characteristics of the type of reaction varied by food. Respiratory symptoms were present in 40% of those who failed tree nut challenges (all of whom received epinephrine), but no patients who failed peanut, milk or egg challenges (P < .001). Subjective reactions (oropharyngeal or behavioural symptoms during ingestion period) were more common in egg and milk challenges than peanut or tree nut challenges (P = .001).

Skin prick testing was positive at initial or subsequent evaluations in 181 patients, and negative in 97 patients overall (SPT not done in 35 patients, who were followed by serial food serum-specific IgEs). Median skin test size was 3.8 mm for egg (range 0–17.5 mm), 5.8 mm for cows’ milk (range 0–12.5 mm), 5.8 mm for peanut (range 0–17.5 mm), and 6.6 mm for cashew (range 3.5–10 mm). Serum food-specific IgEs were performed in 297 patients, and were positive at initial or subsequent evaluations in 147 patients.

Table 3 describes the SPT and specific IgE results of failed versus passed oral challenges. Skin test size was significantly higher in those who failed oral challenges overall (median wheal diameter 6.5 mm vs. 2.0 mm; P < .001). Skin test size was not significantly correlated with challenge failure rate for egg, milk, or tree nut but was significantly correlated for peanut (median wheal diameter 7.5 mm vs. 3.25 mm; P < .001).

**Table 3:** Median skin test and specific IgE results in failed versus passed oral challenges

	Passed	Failed	P value
Skin test size overall (mm)	2.0	6.5	<.001
Skin test size to peanut (mm)	3.25	7.5	<.001
Specific IgE overall (kU/L)	<.35	.70	<.001
Peanut specific IgE (kU/L)	<.35	.78	<.001

Food specific IgE was significantly higher overall in those who failed oral challenges (median .7 kU/L vs < .35 kU/L; P < .001). Food specific IgE level was not significantly correlated with challenge failure for egg, cow’s milk or tree nut, although there was a significant difference for peanut (median .78 kU/L vs < .35 kU/L; P < .0001).

Food dose eliciting a reaction in challenge failures was significantly different (P = .01) between milk, egg, peanut and tree nut, with many peanut and tree nut challenges reacting at low doses, and egg and milk challenges reacting at higher doses. Median final dose ingested prior to an allergic reaction for egg was 2.0 mg, for milk was 3.0 mL, for peanut was .30 mg and for tree nuts was .75 mg. There was no significant correlation between initial reaction characteristics (organ involvement) and reaction characteristics at oral challenge.

There were 5/8 (63%) failed cashew challenges. Cashew was significantly more likely to cause a reaction at oral challenge than the other tree nuts (63% versus 24%; P = .05). Cashew oral challenges were significantly more likely to cause anaphylaxis (P < .001) with a rate of 80% for cashew, compared with 17% overall. Of the cashew challenge failures, 3/5 (60%) had no prior known exposure to cashew, and were avoiding it due to peanut or other tree nut allergy.

## Discussion

Our study shares some findings that are similar to previous studies. Oral challenge failure rate of 33% is in keeping with other studies that have reported challenge failure rates varying from 18.8 to 43% [4–10]. Similar to other studies, we found increased risk of challenge failure in children with asthma and eczema. Perry et al’s retrospective review of 604 oral challenges also noted increased risk in children with eczema or asthma, but not other atopic disease outcomes [9]. Our population, similar to Perry et al’s study, is that of a tertiary care facility which may lead to higher atopic rates than seen in other primary or secondary care settings. Finally, similar to previous studies, we found that skin test sizes and serum food-specific IgE levels were significantly higher for failed than passed oral challenges [6, 9, 11].

Our study had some findings that were discrepant from previous studies on oral challenge outcomes. While the age gap was not wide, older age was a significant risk factor for challenge failure in our population, which is discrepant from Lieberman et al’s findings of no age difference between the group that passed OFCs and the group that failed in their retrospective review [6].

We also found a strong difference in rate of oral challenge failure and severity of reaction based on food allergen. Oral food challenge failures were significantly more common for peanut than they were for milk, egg, or tree nuts (P = .001). To our knowledge, this has not been reported in previous studies. In contrast, Spergel et al’s retrospective review noted milk, egg and peanut to be the most common causes of positive oral challenges, and also the most common cause of multi-organ involvement [10].

There was an overall anaphylaxis rate of 19%, which is higher than some other studies on oral food challenge outcomes [5, 6]. There were no biphasic reactions and no hospital admissions in our study, which has been echoed by other retrospective reviews as well [8]. As with Jarvinen et al’s analysis, our study reveals that anaphylactic reactions were most common for peanut and tree nuts, suggesting that more caution is warranted in performing these challenges [5]. In contrast, Perry et al’s retrospective review found no difference in reaction severity based on which food was challenged [8]. We did not find a correlation between reaction type at presentation and at oral challenge. Some studies have also found no correlation between reaction types [12] although Spergel et al’s did [10].

Our study is unique in its inclusion of tree nuts—many previous retrospective reviews of oral challenges have focused on milk, egg, and peanut [4, 7, 11]. To our surprise, reactions to cashew were both common and severe. It is striking that, of the cashew challenge failures, 60% had no prior known exposure to cashew and were avoiding it due to peanut or other tree nut allergy. The literature on severity of cashew allergy is sparse although a recent systematic review on cashew allergy did note that anaphylactic reactions appear to be very frequent with cashew, and may be more frequent and/or more severe than peanut reactions [13]. To our knowledge this is the first study reporting oral food challenge outcomes on cashew and our results suggest a need for caution when performing an oral challenge to cashew.

There are some findings from our study that, to our knowledge, have not been reported in prior studies on oral challenge outcomes. For example, we report that subjective food challenge failures were high for milk and egg, but not for peanut or tree nuts. The reason for this is unclear but may be partially related to tolerance of the food in question as peanut and tree nut challenges were intermittently masked at the discretion of the attending allergist, often with pudding, while cow’s milk and egg challenges traditionally were not. To our knowledge, this is the first study to stratify based on subjective or objective challenge failures, and the first to report that rate of subjective challenge failures differed by food type. We also found that eliciting dose varied by type of food. Children reacted at low doses to peanut and tree nut (median final dose .30 and .75 mg respectively) while they reacted at higher doses for egg and milk (median final dose 2.0 mg and 3.0 mL respectively). In our study population, children who did not react to the first few doses of peanut or tree nuts tended not to react, while they tended to react later in the protocol for milk and egg. To our knowledge this has not been reported in other studies to date.

There are several limitations to our study. It is retrospective in nature, although most studies on oral challenge outcomes share a similar study design. The challenges were open challenges, instead of double blind placebo controlled challenges, which would be the ‘gold standard’ although are typically not a practical approach. As our center is a tertiary care center, there is a high prevalence of other atopic disease which might make these patients higher risk. As the study was exclusively pediatric, results can only be applied to the pediatric population. While subjective symptoms were included, it is possible these symptoms could be due to anxiety as opposed to clinical reactivity. Some oral challenges to cow’s milk were considered complete at a dose of 30 mL of cow’s milk (approximately 1 g of milk protein) while typically protocols recommend a standard portion of cow’s milk or 10 g solid cow’s milk protein. Some oral challenges were done in children with negative skin prick testing, or had never eaten the food, and it is possible these children were not allergic at baseline, skewing results.

In conclusion, oral challenge failures occurred 33% of the time, and were more severe to peanut and tree nuts than to egg or milk. Children who reacted were older, had higher rates of eczema and asthma, and higher skin test sizes and/or serum specific-IgE levels to the food in question. Eliciting dose varied by food, with children reacting to lower doses of peanut and tree nuts than milk or egg. There was also a high subjective challenge failure rate to egg and milk. Finally, cashew challenges carried a high risk of severe reactivity, even in children with no prior history of cashew ingestion.

## Abbreviations

OFC: oral food challenge
SPT: skin prick test
